# Keys to academic success for under-represented minority young investigators: recommendations from the Research in Academic Pediatrics Initiative on Diversity (RAPID) National Advisory Committee

**DOI:** 10.1186/s12939-019-0995-1

**Published:** 2019-06-18

**Authors:** Glenn Flores, Fernando S. Mendoza, Michael R. DeBaun, Elena Fuentes-Afflick, V. Faye Jones, Jason A. Mendoza, Jean L. Raphael, C. Jason Wang

**Affiliations:** 1 Research Department, Connecticut Children’s Medical Center, and Department of Pediatrics, University of Connecticut School of Medicine, 282 Washington St, Hartford, CT 06106 USA; 2 Department of Pediatrics, Stanford University School of Medicine, and Lucile Salter Packard Children’s Hospital, Medical School Office Bldg., X240, 1265 Welch Road, Stanford, CA 94305 USA; 3 Division of Pediatric Pulmonology and Vanderbilt-Meharry Center for Excellence in Sickle Cell Disease, Vanderbilt University School of Medicine and Vanderbilt Health, 2200 Children’s Way, Ste 11101, Nashville, TN 37232 USA; 4 Department of Pediatrics, University of California, San Francisco, School of Medicine, and Zuckerberg San Francisco General Hospital, 1001 Potrero Ave, San Francisco, CA 94110 USA; 5 Department of Pediatrics, University of Louisville Health Science Center and School of Medicine, 323 East Chestnut, Abell Administration Ctr., Room 502, Louisville, KY 40202 USA; 6 Department of Pediatrics, University of Washington School of Medicine; Seattle Children’s Hospital, and the Fred Hutchinson/University of Washington Cancer Consortium, PO Box 5371, Suite 400, M/S: CW8-6, Seattle, WA 98145 USA; 7 Department of Pediatrics, Baylor College of Medicine, and Center for Child Health Policy and Advocacy, Texas Children’s Hospital, 6701 Fannin Street, Suite 1710, Houston, TX 77030 USA

**Keywords:** Workforce, Diversity, Minority groups, Racism, Discrimination, African Americans, Hispanic Americans

## Abstract

**Background:**

Although Latinos, African-Americans, and American Indians/Alaska Natives comprise 34% of Americans, these under-represented minorities (URMs) account for only 7% of US medical-school faculty. Even when URMs become faculty, they face many substantial challenges to success. Little has been published, however, on keys to academic success for URM young faculty investigators.

**Methods:**

The Research in Academic Pediatrics Initiative on Diversity (RAPID) goal is to enhance the professional advancement of URM junior faculty pursuing research careers in general academic pediatrics. One important RAPID component is the annual mentoring/career-development conference, which targets URM residents, fellows, and junior faculty, and has included 62 URM participants since its 2013 inception. A conference highlight is the panel discussion on keys to academic success for URM young investigators, conducted by the RAPID National Advisory Committee, a diverse group of leading senior researchers. The article aim was to provide a guide to academic success for URM young investigators using the 2018 RAPID Conference panel discussion. A modified Delphi technique was used to provide a systematic approach to obtaining answers to six key questions using an expert panel: the single most important key to success for URM young investigators; ensuring optimal mentorship; how to respond when patients/families say, “I don’t want you to see my child because you are ____”; best strategies for maximizing funding success; how to balance serving on time-consuming committees with enough time to advance research/career objectives; and the single thing you wish someone had told you which would have substantially enhanced your success early on.

**Results/Conclusions:**

This is the first published practical guide on keys to academic success for URM young investigators. Identified keys to success included having multiple mentors, writing prolifically, being tenaciously persistent, having mentors who are invested in you, dealing with families who do not want you to care for their child because of your race/ethnicity by seeking to understand the reasons and debriefing with colleagues, seeking non-traditional funding streams, balancing committee work with having enough time to advance one’s research and career by using these opportunities to generate scholarly products, and asking for all needed resources when negotiating for new jobs.

## Background

Certain racial/ethnic minority groups continue to be under-represented in academic medicine in the US. Although Latinos, African-Americans, American Indians/Alaska Natives, and Native Hawaiians/other Pacific Islanders comprise 34% of the US population [1], these under-represented minorities (URMs) account for only 7% of US medical-school faculty [2]. Even when URMs become US medical-school faculty, they face many substantial challenges to success, including a significantly lower probability of receiving National Institutes of Health (NIH) investigator-initiated research funding [3], about half the likelihood of receiving a mentored NIH K career-development award [4], and obstacles to achieving success in academic medicine, including negotiating for protected research time, how “non-academic” products fit into career development, racism and discrimination, coping with isolation as an URM faculty member, and receiving adequate mentorship [5].

Very little has been published on keys to academic success for URM young investigators in academic medicine. The Academic Pediatric Association Research in Academic Pediatrics Initiative on Diversity (RAPID), funded by the National Institute of Diabetes and Digestive and Kidney Diseases (NIDDK), is a research educational program aimed at successful recruitment, retention, and professional advancement of URM junior faculty in general academic pediatrics who are pursuing research careers [6]. One of the most important RAPID components is the annual mentoring and career-development conference, which targets URM residents, fellows, and junior faculty in general pediatrics, and has included 62 URM young-investigator participants from across the country since its inception in 2013 [7, 8]. A highlight of this highly rated conference is the panel discussion on keys to academic success for URM young investigators, which is conducted by the RAPID National Advisory Committee (NAC) members, who are a diverse group of leading senior researchers in general pediatrics [9]. The objective of this article is to address a major gap in the literature by providing a practical guide on keys to academic success for URM young investigators.

## Methods

The data source is the RAPID Conference panel discussion from the 2018 Conference, which addressed six critical questions: 1) What’s the single most important key to success for a young investigator from a disadvantaged background? 2) How does an URM young investigator ensure the best possible mentorship? 3) When you have a patient or family who says, “I don’t want you to see my child because you are ____,” what do you do? 4) In these very challenging times for obtaining research funding, what are the best strategies for maximizing funding success? 5) As an URM investigator, how do you balance volunteering or being chosen to be on often time-consuming committees with having enough time to advance your own research and career objectives? And 6) What is the single thing that you wish someone had told you, which would have substantially enhanced your success early on?

A modified Delphi technique was used to provide a systematic approach to obtaining answers to these key questions by leveraging a panel of experts [10]. The Delphi technique allows a group of geographically scattered national experts to come together and provide and synthesize knowledge, experience, and expertise on particular topics [10, 11]. The six questions were specifically developed to identify the keys to academic success for URM young investigators. These questions were piloted in 2013 at the First Annual RAPID Mentoring and Career-Development Conference, and further refined during the subsequent four conferences, based on NAC responses. After the completion of the 2018 panel, the session transcript was provided to all NAC participants in the second round of the Delphi technique. NAC participants then provided feedback on or modifications of their responses to the panel questions, and all NAC participants reviewed the entire session to facilitate consensus. Consistent with recommendations for the Delphi procedure [10], the NAC panelists consisted of a heterogeneous group of experts representing both genders, multiple races/ethnicities, and the spectrum of geographic diversity in the US. Additional details on the NAC members are available elsewhere [9].

To summarize the overarching themes of responses to each of the six questions, grounded theory was used to perform a thematic analysis and create a thematic taxonomy.

## Results: RAPID conference panel findings: keys to academic success for URM young investigators

The overarching themes of the expert panel’s responses are summarized in Table 1. The following sections provide the specific, detailed responses of each panel member to the six key questions.

**Table 1:** Taxonomy of themes in the responses by National Advisory Committee members of the Research in Academic Pediatrics Initiative on Diversity to six questions on the keys to academic success for under-represented minority young investigators

Question	Response Themes
What’s the single most important key to success for a young investigator from a disadvantaged background?	• Having mentorship • Having multiple mentors • Write often • Be strategic about your financial situation • Negotiate well, especially regarding protected time for research • Be passionate about what you study • Get the right training and be confident about your ability to achieve your goals • Don’t view yourself as “disadvantaged” • Be tenaciously persistent and “fail productively” (learn from each failure)
How does a young investigator ensure the very best possible mentorship?	• Assemble several different mentors to address your multiple needs • Don’t be passive: work hard to get what you need from your mentors • Identify your future professional trajectory • Ensure that your mentor has a proven track record for success • Find a mentor who will invest in you • Spend sufficient time with your mentors • Make sure that your personality/style is a good fit with the mentor • Have a mentor who also is a sponsor, with a clear investment in you and your success • Have at least one mentor who is a “coach”: able to provide honest feedback, assist with achieving work/life balance, and facilitate networking
When you have a patient or family who says, “I don’t want you to see my child because you are ____,” What do you do? How do you react so that the family gets the best care and you’re being ethical, while protecting yourself?	• Seek to understand the reasons • Exercise professional detachment • Help the patient nevertheless • Seek training about unconscious bias • If the patient is medically stable, transfer her/him • Hold open forums to discuss incidents and develop coping strategies • Protect and take care of yourself • Debrief and process with trusted colleagues
In these really challenging times for obtaining research funding, what are the best strategies for maximizing funding success?	• Use small grants to build your portfolio • Seek non-traditional funding streams • Leverage funding from mentors • Write many proposals • Collaborate with partners • Network • Strategically analyze the funder pay lines • Establish a publication track record • Negotiate protected time and resources in new jobs • Participate in study sections
How does an under-represented minority balance volunteering or being chosen to be on often time-consuming committees which, of course, need diverse voices like yours, with having enough time to advance your own research and career objectives?	• Using these opportunities to generate scholarly products • Add such committee work to your CV and turn it into scholarly products • Choose committees strategically with the help of a mentor • Join committees in the context of being an expert • Developing a triage process based on interests and being politically savvy • Balance commitments with an eye towards promotion • Prioritize activities that promote diversity and excellence
What is the single thing that you wish someone had told you when you were a beginning young investigator which would have substantially enhanced your success early on?	• Ask for all needed resources • Identify what others negotiated for to determine the components of competitive job offers • Be focused • Be open and seek advice • Get outside your comfort zone • Learn how to write well and often • Focus on fulfilling your own standards of excellence, rather than achieving external milestones and validation

*G. Flores:* **What’s the single most important key to success for a young investigator from a disadvantaged background?**

*F. Jones:* For me, the most important thing was having mentors. Not just one, but multiple mentors. I didn’t plan on doing academics when I first began my career. I was in Milwaukee, doing a public-health loan obligation, it was cold [laughter], the university offered me a job back at Louisville, and I said, “okay.” So I had no idea what I was doing. What got me through and kept me going is mentorship. I still use multiple mentors today, and I think that is exceptionally important that you find mentors. Not necessarily one that’s assigned to you. Pick them out, what you think you need.

*J. Raphael:* Mentorship would have been my choice as well. The other thing is write, write, write. You should be writing constantly. It should be your default. You should be constantly writing, because that’s the way that you get into the habit of being scholarly, and the more you do that, the easier it becomes, the easier you can get products out the door. And then it becomes such a great habit: if you’re not writing, you start to wonder, “Is this a good use of my time, because I know that career success is going to be based on how well I write and get these products out in a number of different ways. If I’m not doing that and I’m not doing clinical work - because obviously that is important as well - why am I doing whatever I’m doing?” And that helped me, because it was advice somebody gave me, and I first thought, “I don’t know how this is going to help me.” But over time, I started to feel that tension. If I wasn’t writing, I really started wondering, “Is this a good allocation of my time?” So that really just helped me prioritize and think about where all my efforts should be, and it made me a lot more productive in the end.

*J. Wang:* I’m going to say something slightly different. Perhaps not the most important factor, but definitely important, is early on, you have to be strategic about your financial situation, because a lot of people can’t afford to go into academics because of their financial situation. So it’s good to plan early and make some really strategic decisions about savings and investments, so that you won’t end up with a lot of debt. People probably don’t tell you that, but a lot of our decision-making is very simple. It’s based on financial outcomes and it’s no different in this group and many groups. It’s not the most important consideration, but I thought I’d throw it out there for people to consider.

I was going to choose mentorship, too, but besides mentorship and writing all the time, one thing you need is protected time, and a way to get that is negotiating well. When you approach your first faculty position and subsequent ones, make sure you have adequate protected time to do your scholarly work, whether it’s education or research, and you always have to account for your clinical time spilling over by 10, 20, 30% into your protected time, because that’s just how it goes, and so account for that. So, a 50% research position is not really 50%, it’s a lot less. Aim higher than that, if you can.

*F. Mendoza:* Two things: First, be passionate about what you want to study. If you are passionate about a topic, then studying will be more enjoyable, and thereby more fun than work. I say this only because any academic pursuit will be time-consuming, and one needs to have a short-term gain. Second, determine what you need to do to have confidence in your work and know that you are capable in your field of study. This usually means having the appropriate training in the field and bringing yourself to its cutting edge. By this I mean that you know more about your topic than anyone else, because you have read the literature, linked with others in the field, and thought about the important questions in your field. All of us on this panel believe that all of you are capable of achieving this goal.

*M. DeBaun:* I agree with everyone on the panel. I also think it’s imperative to have a frame of mind that doesn’t put you in the “box” of being disadvantaged. I’m not even sure what that means. Fernando and I had this conversation last year; I never grew up thinking I was disadvantaged. I still don’t think I’m disadvantaged; I was not raised to believe I am disadvantaged. I refuse to embrace this concept that I am or was “less than.” I believe that the single most important key step is to believe that you have the same capacity to be successful as anyone else.

*G. Flores:* I would say the single most important thing is to be tenaciously persistent while failing productively, and by that I mean, Winston Churchill once said that, “Success is going from failure to failure without loss of enthusiasm.” You have to learn to have a very tough skin. You need to be productive with each failure. If you get a manuscript rejected, use that as free advice about how to make your next submission better. Same thing with a grant: If you don’t get an award one year, put it in again the next year and figure out a way to make it better. We’re sitting right here in Silicon Valley, where having failures is actually a badge of success. People here say, “Yeah. Let me tell you about my failures.” Because as Thomas Edison said, “ I have not failed. I’ve just found 10,000 ways that won’t work.” You have to have that mindset to survive, and it’s really about survival.

*G. Flores:* **How does a young investigator ensure the very best possible mentorship?**

*J. Raphael:* One of the most important things is there’s not a one-size-fits-all mentor. A lot of junior faculty struggle with this, as I did. You want to find that person that covers all bases. They are going to be your content expert. They are going to be the person who fits how you think about the world or how you orient yourself to the world. They’re going to understand your methods and everything else. But if you do that, you shortchange yourself, because it’s nearly impossible to find that person. People struggle and get frustrated within their institution because they can’t find this amazing perfect mentor. What you learn over time is that there are different mentors who can address different areas. One may be great for research methods, one for content, one for career. You really have to decide how you want that layout to be so it more becomes a committee of mentors. The other thing is you want mentors in your own institution, but outside your institution as well. Because your institutional mentors have a little conflict of interest when it comes to certain things. They may be a resource when you’re looking for another job and talk with other people in the institution. So it’s always nice to have people external to your institution because those folks can look out for you in a different way. They may have a conflict of interest as well because, certainly, they may say, “Why won’t you come here” [laughter]. But that’s a good thing [laughter]. Try to accept the fact that there is not one perfect mentor, and you need a diverse array of people, internal and external.

*F. Jones:* You can’t be passive, because the mentor-mentee relationship is an act of engagement. It takes a lot of work. Have an idea of what you want from that person, and have a contract, such as, “I would like for you to do this, I will be doing this.” Make sure it’s a relationship, or you may not get as far as you like. It also depends on the trajectory that you want. You have to know what trajectory is best for you. If you say, “I want to take the academic route. I want to work in an academic center, I want to be promoted in the clinician-educator or tenure track,” then there are clearly components of a mentor that you have to have. To put it simply, you need to be on PubMed to see whether a potential mentor has published any manuscripts in the last year. If not, then coming to their lab anticipating you’re going to have six publications in the next year or two is a non-starter. Because you’re not going to get that person to all of a sudden be productive. That’s not the way it works. Then, even if you see that they have 10, 12 publications a year, you want to drill down on, who’s first and last author, because the mentor who’s last on every publication is a signal. And the mentor who doesn’t have fellows or junior faculty who are first authors is another signal. So you really want to take a look at that person’s track record. Last but not least, talk to people in the lab. If they don’t plan to meet with you on a routine basis, like weekly or biweekly, that’s not necessarily an environment that you’ll thrive in, particularly when you’re junior and you haven’t written your first manuscript because you need that oversight and support.

If you want to get a grant, there are NIH K awards, institutional K awards, and foundation grants. Almost all award committees choose awardees, in part, based on the mentor. So if the mentor doesn’t have a track record of awardees who’ve been funded and doesn’t have any funding themselves, be it HRSA, R01, NIH, or foundation funding, then the likelihood of you being selected is nil, because they’re going up against mentors who have a long funding track record. If you have a great mentor or junior person who doesn’t have that track record, the suggestion is that mentor pairs up with a senior mentor, and together, they write your letter of support. Because we all were junior at one point. But it is a matter of making sure that you understand what are the rules of the game. And those are the rules of the game if you decide to go the academic route and pursue the funding ladder of institutional K, NIH K, or foundation award, and then ultimately, independent investigator.

*E. Fuentes-Afflick:* The most important attribute is finding someone who will invest in you. I wouldn’t get personally caught up in what department they’re from. I’ve been at UCSF since I was an intern, and when I was seeking research mentorship as a junior faculty member, my mentors came from internal medicine and OB. I first did the rounds in pediatrics, but didn’t really find anyone who had enough overlap or interest in mentoring me. So I found two people in other fields. One is Dr. Eliseo Pérez-Stable, now the Director of the National Institute for Minority Health and Health Disparities. The other was Dr. A. Eugene Washington, an OB who’s now in a senior leadership position at Duke. Both had overlapping interest in what we now call health disparities. We didn’t use that term at the time. But their interests overlapped with mine, and that was our shared bridge. It wasn’t because we were from the same clinical discipline or worked in the same hospital or anything like that. So, I would really encourage you to find someone who will invest time in you and have enough of a shared interest to make the relationship work, but not think it should be this or that kind of person, because, really, the interpersonal relationship is most important. And everything that Michael said about the importance of choosing that person is true, because a lot rides on that, but if you’ve identified a mentor with a great name, but who doesn’t really care about you, that also won’t work. It has to be the mix of the right skills and interest in mentoring you.

*J. Wang:* I totally agree with Elena: Caring is the most important mentor attribute. The other thing is, if you have more than one mentor, think about their roles. Some people are better at operations: helping you to write a grant, editing, and giving you concrete comments. Some people are better at strategy: overall vision and career planning. Some people are better at enlightenment: work/life balance is very important. You need all these things to be successful and have both a good career and life. You want mentors that offer enlightenment, strategy, and operations, and they might not be in the same person.

*J. Mendoza:* Make sure you spend a lot of time with your mentor and mentor committee. Make sure your personalities click, that you actually look forward to spending time with your mentor [laughter], or at least look forward to getting feedback from your mentor and making plans. You’re not going to become best friends, of course, but you have to be able to have a good working relationship. Even among some of the most successful scholars, sometimes, personalities just don’t work out. So don’t force it just because they’ve published like 500 papers and have 10 NIH grants. It’s not worth the conflict [laughter].

*F. Mendoza:* Do you know the difference between a mentor and a sponsor? Raise your hand if you do. What is it? Answer: A mentor is someone who provides personal and professional support in your development; a sponsor is someone who goes beyond this and tries to connect you to other leaders in the field in order to help you navigate your career path to success.

We talk about mentors, and everybody says that’s important. But as you heard, there are a lot of different types of mentors. Clearly, you need to know what areas you need help in, and to address those with mentors. Sometimes, mentors can address more than one area. It may be methodology, it may be career development, it may be how you balance life, etc. But you also need to have somebody that says, “I’m going to invest in you.” That’s a sponsor. A sponsor is somebody that has, as they say, skin in the game. The skin in the game is if you’re successful, they’re successful. There are a lot of mentors that mentor just because that’s their job to do in the department. But they have no skin in your game. They can say, “I mentored them, and it’s their problem if they don’t get a job.” Or, “it’s their problem that they don’t publish. Their lack of academic success is their problem; they just didn’t have it.” So, having a sponsor is key. A sponsor has to have an invested relationship with you that goes beyond a typical mentorship relationship. This invested relationship is what adds to their lives. Basically, the relationship between the sponsor and mentee is that the sponsor is going to add to your life by making sure you’re successful. And you’re going to add to their lives by having them feel good because they made you successful.

*G. Flores:* I would add that you want at least one member of your mentoring team to be a coach, a supreme coach in the true sense of the word. What does that mean? First, that they’ll be blunt with you when you need some bluntness about, let’s say, your research really isn’t up to snuff. Because even though that might initially hurt your feelings, it, in the end, will actually help you. They also should be somebody who can help you balance your work and personal life, as we often, in academia, ignore the latter. And if you don’t have balance, then it’ll be difficult for you to be productive. And one of the most important things, and I maybe didn’t learn it well enough until later on, is make sure that that person opens doors for you in terms of networking, whom do you need to know, who are the key people in your field. Can they connect you with somebody who can help you with a grant, a publication, your K-award mentoring team, or a future collaborator? That’s just so invaluable.

*G. Flores:* It’s been decades since we’ve seen this level of racism, anti-immigrant sentiment, and bias in the US, and it goes all the way to the leadership of our country. It’s something you can’t avoid in daily affairs, and I’ve recently come across a few articles about physicians who have to confront racism with their patients and families. **When you have a patient or family who says, “I don’t want you to see my child because you are ____,” what do you do? How do you react so that the family gets the best care and you’re being ethical, while protecting yourself?** Because that can be a really traumatic experience.

*M. DeBaun:* I just want to tell a story that addresses this point. I went to see a family of a new patient who had just moved in from Alabama to Nashville, and the mother looked like something inappropriate had happened. She was very closed mouthed, arms crossed, one-word answers, and it was an odd interaction for a first encounter with a family. Our clinic then had a family retreat four or five months later, and I invited the mother, and made sure that she came to the family retreat with her daughter. And she didn’t think I remembered her, but I remembered her. I walked over to her, gave her a hug, and said, “glad you’re here,” and then, “Remember that time I saw you four months ago, and you were not talkative, and you didn’t really respond? What happened?” She replied, “I’ve never had anybody black in charge. I didn’t know if you were legitimate or not” [laughter]. So, there is this idea that this oppressive thought process only comes from the majority, but it can also happen in the other direction, and so I carry both with about the same weight. I seek to understand, and then I move on, because, at the end of the day, I know I have a gift to bring somebody when I provide medical care for their child, adolescent, or adult, and they can make a conscious decision to go elsewhere. That’s their call, and I’m okay with them choosing to seek care elsewhere. So if there happened to be a white guy with a swastika on his forehead, and he has that same reaction, I’m going to come in the room with the same posture that I did for this mother, and then I’m going to ask him, maybe not in that initial visit, but eventually, I’m going to say, “What gives? Why do you have this anger? Is it something I did? You had a bad day, but I want to understand.” So I don’t see the two extremes differently. I react in the same way.

*F. Mendoza:* When I was a young faculty member, a first-year attending, there was a family who came in with a child with asthma. They said, “I don’t want a Mexican doctor taking care of my baby.” And I said, “Well, okay. Let’s make sure your child gets good care.” But it was funny, because it didn’t make me feel less, because I knew, as Michael was saying, I’m a good doctor. I’m the attending on the pediatric ward at Stanford, well trained. In the final analysis, as Michael points out, we’re professionals. What does it mean to be a professional? It means that you’re an expert, you know you’re an expert, and you deal with a patient as a patient, not just the dynamics of the physiology, but the dynamics of the background, politics and all. That’s part of taking care of patients. Professional detachment is useful in dealing with a situation like this. At the same time, you can sometimes feel sorry for them and their bigotry, but your professional role is to help the child, and if they see that, sometimes they apologize, and they change. We’ve just finished some e-modules for training on unconscious bias, both directed at patients, and for patient bias toward physicians. It’s important for us to start thinking about how to train ourselves to deal with the various forms of unconscious bias. Because at the end of the day, half of all kids in this country are minority, and we’re seeing racism and other “-isms” out there. It’s incumbent upon us not to degrade ourselves by moving down to that level of bigotry, and to stand up here as professionals, to say, “Look, we’re doctors. We’re well trained. We’re here to help.” “If patients don’t want our care, then they have the option to go someplace else.” At our hospitals, if patients are hostile toward physicians or staff, they can be transferred, if medically stable. Our hospital's leadership have shown that they support a respectful work environment for our staff. Unfortunately, in our current environment, we will need to deal with this, but we, as leaders, need to stay professional, and we need to show the light of caring. Martin Luther King said darkness doesn’t wipe out darkness. Light wipes out darkness, and I think we’re the light.

*F. Jones:* There have been a lot of articles about this issue recently, and it does bring back memories of when I was training. When I was doing my residency, this family was adamant that they did not want me touching their child, but my attending was there. He was a really tall, white guy, former army, and his language was very army-like. He spoke up for me. He was there to make sure things were okay. So I remember that. For him to speak up, it made such a big difference, and I make sure I do that when I hear this same language. Because I’ve gone into a room with another doctor, and they say hello to the “doctor,” and tell me, you can pick up my tray. But what you have to do, like Fernando said, is be professional. Your goal is to treat the child, do the best you can, and then you can deal with the other issues later, but it’s nice to have an outlet where you can come out with it and talk. At the University of Louisville, we provide open forums where we’ll discuss these incidents and figure out strategies on how to deal with them. It gives people an opportunity to express how they’re feeling. It’s relief, and that’s what’s needed.

*G. Flores:* When I was in college, I took an ethics course, and the one thing that stuck with me is, how do you decide what’s the right thing to do? Basically, it comes down to whether a neutral third-party observer floating over the situation would determine that the action that you took was the correct choice. So, the first thing to do is what Michael was saying: see if you can understand why. You may not get a helpful response, but at least you can see if there’s something that you can discuss and have a dialogue about. Second, you need to make sure that the patient gets the care that that patient needs; sometimes children are innocent bystanders. I’ve heard two schools of thought, and I’m not sure which is the right way. In some cases, some people believe that the family who made the racist remark should leave the medical facility, as long as the patient is not gravely ill, and go elsewhere, because that’s not a philosophy that the institution supports. Other people will say, “I’ll go get a provider that you’ll be more comfortable with.” Third, you need to “treat yourself” in that situation. By that I mean, you need to process it and reflect about it. Some people will write about it and then hopefully turn it into a positive by teaching others, by sharing it with your colleagues, by discussing how you deal with it, and then producing a curriculum that we can provide people with like yourself so that when this does come up, you have a clear path. Because it’s easy to get angry, upset, or hurt in that situation. What we want is what a neutral observer would say was the right thing, that at the end of the day, no matter how upset you are, that you feel like you did the best as a human being in that very difficult situation.

*M. DeBaun:* You don’t want to respond to ignorance with feelings of insecurity. When I started out, one response to those situations was to ask the individual, what medical school did you go to? That’s not necessarily going to fight it or do anything -- now you’re becoming insecure. Or sometimes you start to change how you talk in response, and you say, “I’m going to talk like an old person. I’m going to talk in this specific way,” and you try to do all these things to overcompensate in the moment, but that’s not the right approach. It’s what everyone’s saying: you want to stay in that professional role. If you start to let yourself be affected, then you’re not acting in the way that you typically do, and now the child’s not getting the care that they should, because it becomes more about you and trying to respond to that ignorance in a way that takes you away from your professional role and who you actually are.

*G. Flores:* I also want to make sure we address gender issues, because even after we deal with the racism and anti-immigrant sentiment, another current trend is women saying, “We’ve had enough of this, so let’s assert our rights and make sure that we’re heard.” If our panel members have thoughts about how to address gender bias when it comes up in patient encounters, we’d welcome your thoughts.

*E. Fuentes-Afflick:* In medicine, we’re really privileged to have so many opportunities to grapple with those issues, because when you deal with people every day, you are a person, you work with a team of people, and then you are interacting with people, and we’re often interacting in a crisis or really difficult situations, which tend to highlight any shortcomings we have. For me, one of the most important issues is to try to be present in the moment, deal with what you need to do, but if you are picking up on something, or someone says something, handle the clinical situation, but take time during that same day to debrief and process what happened, and consider sharing with a trusted colleague, “This is what happened, this is my interpretation. Do you agree? Do you see anything else?” Because the more we can deal with bias or differential treatment in real time, it’s always going to be better than accumulation and then eruption. It’s also important to recognize that we all have our sensitive points, and someone might say something and it may be intended in the way that you interpret it, or it may have been intended in a different way, if you look at it from a slightly different perspective. Not, of course, every statement falls into that category, but sometimes that does happen. If you have trusted colleagues with whom you can debrief and discuss and then make a plan of correction or a plan to move forward, that’s always helpful. I work at an institution where we’ve got all signs everywhere, all races, all colors, all this, all that. But you can never control what happens between two people or in an interpersonal setting, and sometimes, things happen even with all of that signage. I think it is our responsibility; we cannot walk away from a clinical issue, even if the person is very upsetting to us, but we do need to figure out how to deal with that after the crisis has passed.

*F. Mendoza:* Changing culture occurs because we recognize something and we talk about it, not just those of us of color, but everybody talks about it. You are in positions where you’re supervising residents and medical students. The lowest person on the totem pole is a medical student, and they sometimes come to us as deans and say, “This happened and nobody said anything.” I interview internship applicants, and ask them if they have experienced these kinds of things. Our culture right now doesn’t address this; it sweeps it under the rug and leaves it up to the individual. If we want our hospitals to be better, we need to talk and teach about these issues. We need to allow trainees and colleagues to say, “Here’s what’s happened.” Or you may want to be preventive by saying, “Sometimes, on a clerkship, this may happen. Here’s what I want you to do.” That’s the preventive and proactive way of saying, “Our culture is a culture of acceptance, professionalism, and focus on kids’ health, and here’s how we’re going to accomplish this goal.”

*G. Flores:* For the next question, we’ll “switch gears.” **In these really challenging times for obtaining research funding, what are the best strategies for maximizing funding success?**

*J. Wang:* Since we’re in Silicon Valley, I’m going to talk about some strategies that people use here for startups, and an academic career is no different than a startup. If you think that way, how do you try to build a career, and get the initial pot of money, and grow that money? Traditional sources of funding are drying up. So it’s appropriate to use the word, “bootstrapping.” You know how a lot of companies here do startups; they initially have some idea, and they’re still working for other people, but they try to have allocated time when they focus on what they want to do while still working for the company. And then, they try to bootstrap a little bit of money here and there, and try to build from small projects. Right now, it’s so hard to get an academic job right out of training. So maybe you need to bootstrap and focus on one or two key ideas and apply for small grants, and slowly build that up into a body of work with which you could convince people to invest in you a bigger pot of money, which is no different than a startup. It’s like lean startup. You hit certain milestones, people invest more in you, and then you hit another milestone, and people invest more. I’d be a little entrepreneurial at this moment.

*M. DeBaun:* I didn’t know this at your stage, until I got to my stage and understood the game. This strategy that I mentioned earlier about looking at the funding record of your mentors is important, because they have discretionary funds. So, when you find a mentor that meets all the criteria that we talked about - compatibility, and so forth – but, also, the funding, then they can pay for the statistician that you don’t have the money to pay for. They can send you to New York to give a talk and meet with other people in the same sphere, and you don’t have to say, “I can’t afford to go.” They can take you on a trip abroad to work with the colleagues who are international experts. These are all the intangibles that I saw with my eyes wide open when I became a professor, but didn’t know when I was an assistant professor or a fellow. So, it’s not just about choosing the mentor who has funding, but the funded mentor willing to invest and be a sponsor. It’s a very important component: having access to resources through your mentor. When the K-award committee makes selections, the reason they want your mentor to be funded is because it’s been worked out for several decades that this is how junior people become successful. It’s because their mentor has been willing to invest in them, and they specifically state that there should be resources that the mentor will augment for the mentee’s career trajectory in their scientific proposal.

At the same time, you have to write. I don’t mean just manuscripts; you really have to write grants. If you’re not writing a minimum of three to four grants a year—I couldn’t care less if it’s to the local Presbyterian church down the street that’s interested in health disparities—you have to write. Fernando and I had this wonderful conversation yesterday, about how, after submitting so many grants that did not get funded, we used those as learning opportunities, and the light bulb finally turned on for both of us, like, “hey, I’ve now had my 100th grant rejection, my 101st got funded, and I feel like I know how to do this.” So there’s no shortcut to hard work. You really do have to fail a lot. You really do have to write a lot, and so set a bar and just write and write grants. Not just manuscripts.

*F. Jones:* I’m going to be out of the box because I don’t usually go for NIH funding. Because my work is more community- and diversity-related, so you think about who’s interested in that and how to put things together: in a professional, totally dynamic team that may not have worked together before, from multiple disciplines that will affect the community. So looking at opportunities in that way. That may be through city government, state government, or foundations. You look at those kinds of things and cultivate those relationships. I do like to work, when possible, with a grant writer. We don’t always have that available and we have to do it ourselves, but if you can get that input, it’s exceptional. In our office, we have a small group of people, but lots of partners. And that’s how we put things together: through partners, and figuring out how we can put a proposal together to get the things that we want.

*J. Raphael:* Another important thing is maintaining all these collaborations that you have, either at the local or national level, because those may be the people who say, “you’d make a great person to have on our grant and provide this type of expertise.” So in addition to having that phenomenal resource mentor, look at other people in networks who may be doing things. That means getting out there at your typical meetings at the hospital, in medicine, and at conferences. Because those opportunities will come up. One thing to balance is, if you’re going to be on someone else’s grant, you have to think about “what is the minimum effort you need to be productive?” Because Jay and I were talking about this “death by 5%” earlier [laughter]. It’s that not all funding is good funding, in the sense that sometimes you might get a small piece. But then that person may ask you to do a lot more than that. So now you’re really tied up with a little bit here, a little bit there. You have your biweekly meetings for those grants, and before you know it, you don’t have your own career, your own thing, your priority. So you want to go after where the funds are, but in a methodical way where you still can achieve success and conduct the research you want to do. Because there is going over the edge, where you just have all these little things, but then they’re outstripping the time that you actually have.

*J. Mendoza:* I have some more practical advice. For those of you who are going to become clinician-scientists, you’ll be looking to the NIH for a K award. It would be wise to examine the pay lines for K awards for the various NIH institutes that you are considering, since some have more favorable pay lines than others, as well as larger budgets. So keep that in mind. Many of our colleagues have built their careers on obtaining funding from smaller institutes, and they do great. It can be more difficult to crack that egg now, because funding is so tight. For example, I have obtained funding from a variety of NIH institutes for my research on inequities in youth physical activity and nutrition. For those of you looking to bridge your way to a K award, go for those small grants. Also look at diversity supplements for your mentor's existing R01 grants. There are creative ways to build your career up to that K Award. And once you get that K Award, it’s kind of like a golden period where you have four or five years of protected time. You may not write three or four grants every year during that K award because you’ll have to actually do what you said in the K Award. So that will be a unique time in your career where you’re not writing as many grants. Once the K Award is halfway finished, you will be writing three to four grants yearly. And that’ll just kind of be of how your career goes, unless the golden age of NIH funding comes back to us [laughter], which doesn’t seem likely.

*M. DeBaun:* Jason said something that’s not intuitive, and I didn’t know what Jason just said when I was at your stage. You can go to the NIH website and look at the funding for each institute. Within the institutes, they tell you how many grants they give for each funding level. The K type Award (mentored award) is a special funding pool. For junior faculty in this room, it would be a K23, so you would look specifically for how many K23 s were awarded by each institute. You really have to spend some time looking at the data to have a handle on what was just said. And within institutes, they have a pay scale. They tell you, “we fund at the 15th percentile,” or “we fund at the 20th percentile.” Last, but not least, the best time to write a grant is when you don’t need it. Don’t wait until Year 4 of your K award to write your first grant since you received your K23 award, because there’s an acquired skill to writing successful grants. So, I respectfully would say, write as soon as you can.

*E. Fuentes-Afflick:* Remember that societies and other organizations often have travel awards. They have Young Investigator Awards. They have different categories of awards, and even if the amount is small-- as Jason was saying, bootstrapping, right, a little this, a little of that. What Jay said is important, because “a little bit” is not always a little bit of work. But keep your eyes open for those kinds of opportunities, especially for travel and presentation at conferences. Many institutions also have internal travel awards. If you have an abstract accepted, then you’re eligible, perhaps, to apply for funds that will help support your travel. The only caveat I would add to what Michael said about mentors having discretionary funds is that everyone is feeling the squeeze, including mentors. So that’s often true, but maybe not as true as you would want it to be. Just keep an open mind about all the various sources of funding. The other point that Jean made that’s really important is networking. Invest in the relationships that you have here today. It certainly happens for us at the mentor or faculty level, because people come in and out of your life in ways that will surprise you, and the last thing you want is for someone to remember you as rude, arrogant, or not dependable, because that will haunt you for a long time. But if you show up, you’re collegial, “Hey, how you doing?”—that’s the kind of relationship and reputation that will definitely help you.

*F. Mendoza:* Make sure you connect with Elena. She’s the president of the American Pediatric Society, which is the senior society for academic pediatrics. Let me bring this together a little bit. I’m on the Advisory Council for the National Institute on Minority Health and Health Disparities. All these councils want successful minority candidates. What does that mean? Certainly, all the things you heard is important. But at the end of the day, you have to show that you’re a scholar. What I tell my mentees is, if you write one paper, that’s great. If you write a second paper on that topic, people note, “you wrote a second paper.” If you write three papers on the same topic, then you become an expert. At the end of the day, it’s important to focus on what’s important to you, but also know how to write and get published. If you submit a grant for which you can show these types of accomplishment, three papers on the same topic, a focused research topic, access to good mentorship/collaborators, you’re more likely to get funded.

*G. Flores:* I would add that you want to set yourself up from the beginning for success. By that, I mean, when you negotiate for your job, get as much protected time as you can. Get seed money; it’s perfectly reasonable to ask for some funds upfront to do your research. Make sure you have, as was mentioned, at least one of your mentors who has a solid, established NIH or AHRQ funding record. One of the best ways to learn how to write a grant is to be on a study section. And that can be internally. It can be NIH; sometimes they’re looking for young investigators, particularly with some diversity. And, as mentioned earlier, look for other bridging grant sources. Of course, the RAPID award is a great one. And then I want to remind you I’m on the National Advisory Committee for the Harold Amos Medical Faculty Development Program, which will consider every kind of research, from basic science on up to health services research. Dr. Debaun, I believe, is an alumnus, as am I. And you can hold one of those at the same time as a K award. So have a look at AMFDP.org.

*E. Fuentes-Afflick:* You’ll notice from the Pediatric Academic Societies or the Academic Pediatric Association, there’ll be calls for abstract or workshop reviewers. Even if you think, “ I don’t have a lot of expertise,” you can request a specific area you want to review in. And it also gives you a bigger sense of how people write, and how you fit it into that abstract box. Now you’re scoring and contributing to the quality of the meeting, but that’s also another way to get a toehold into this reviewing-as-learning process. And it’s hard to get onto a study section or grant review process until you have a little more of a track record. But the lower-stakes abstract review is really easy to just sign up for and be a part of.

*G. Flores:* Next question: **how does an under-represented minority balance volunteering or being chosen to be on often time-consuming committees which, of course, need diverse voices like yours, with having enough time to advance your own research and career objectives?**

*F. Jones:* Being black *and* a woman, I count for two things, and I’ve been told that when I get put on committees. You’re two marks that they can check off. And it’s amazing how many committees and everything else that people want me on. So what I have done in order to accommodate the things that I like and do all these other academic activities as expected of me, I use that for some of my scholarly activity. Using the things that happen with those committees or things that we want to do, we can write about it, and do something with it, and use it as scholarly activity. So it helps balance things out a little bit.

*E. Fuentes-Afflick:* If you think that your perspective is important, or you represent some bigger group, and you think that that perspective is not well-represented around the suites of power, you have to show up. You have to talk, you have to sit in the chair, and you have to do the work to represent that. So it’s not fair to think it’s someone else’s responsibility to represent that perspective because you’re too busy doing other things. So I like the approach that Faye raised in terms of converting that experience into something scholarly, into something more. Although, make sure that your CV is your friend. We have an online system at UCSF. Everything that you do, put it on your CV. You’re mentoring someone, you’re attending this conference, put that on your CV in terms of national conferences that you’ve attended. Everything needs to count, because if you try to update it once a year, you’ll forget things. But if you do it as you go, then it’s easier. But I feel like there’s no substitute for participation and showing up. In some ways, it becomes a virtual circle, because then you have a reputation for being involved and sharing a perspective, and that can lead to other things. It may not be what you thought it was going to lead to, but it does help, and as scholarly as you can make it along the way will be to your benefit.

*J. Mendoza:* I’ll go back to my early career days, when you struggle to just exist, like you need to justify your existence at the institution. You need to get that K award or whatever it is that you need to get. So you’ve entered academia, you still need to do service. The thing you can be strategic about it is, with your mentor or your mentoring committee, you can pick and choose what you take on, and going into it, you should know how many hours of meetings are needed and how much work is required outside of those meetings, because, again, that can bleed into your protected time. And once you reach associate professor, once you become more well ensconced and feel more comfortable at your institution, then you can really shine, get out there, and serve on multiple different committees, if that’s your desire and where your heart is. But when you’re early-career faculty, you’re at a very vulnerable time, and so I would be strategic. Yes, you need to serve, but it shouldn’t overtake the rest of your academic time.

*J. Raphael:* It’s such a tough situation, because when you start at the institution, you want to contribute to all these different ways. Some people call this the minority tax. It’s tough because, sometimes, you do these activities that are more along the lines of volunteerism; it may feel good to talk to a school from 9:00–10:30, but after you’ve done that four times, that’s a lot of papers and grants that didn’t get written. Then you feel guilty and start thinking, “What was the impact of what I did? What was really good?” So what I came up with over time for myself is, I do want to be that person at the table, but I’d like to do it in light of being an expert, because you have more credibility that way. Because if you just become the minority person on all these committees, you won’t get the respect, because you don’t bring expertise to the table if you’re not accomplished in some way. If you become that amazing medical educator, you can speak in a way that no one else can. If you become that amazing researcher, your words are taken in a different light. For me, it was go out, develop your track record, and then you can speak. Once you get to that point where you become an expert, you don’t just have to be the minority person at the table. You’re actually in a completely different position, because you’ve earned it in a different way, and people are going to respect more what you have to say. It’s not like this is a person we put on all these committees. This is the person who is now the Dean for Research, the Vice Chair. All the accomplished people here, they’re doing it in those different facets. They’re not doing just because they have to. If they do it, they’re doing it in a very vigorous way where they’ve done the training and have the time allocated. They’re not just doing it without thinking of strategy. It’s a hard thing that you struggle with as junior faculty. What I would always say is just go back to what your expertise is. Think about what you want to be, and come to the table as that person, not just the person who’s being the activist for the institution, because if you are, I just don’t think you get as much respect.

*G. Flores:* Develop a triage process. Is it something that you’re really interested in? Because if you have a passion for it, then, by all means, do it. There are some requests that you have to do. If you’re department chair or division chief or the dean says, “Camille, I’d really like you to be on this committee,” you have to make sure that you are politically savvy and do things that people feel you should be doing. And then there may be situations where you feel like you can’t do it. At a junior level, the smartest thing to do is not just to say, “no.” It’s also helpful to go to that good mentoring team that you have and say, “I don’t know if I can do all this. What do you think?” And hopefully, they can run interference for you or tell you a way to navigate through the system.

*F. Mendoza:* One should always strive to obtain a balance of participation in “minority” activities and doing your academic work—but a balance with the goals of making sure you get promoted to associate professor and that you are an academic leader. When I started in 1981, there were 0.5% Mexican-American faculty in the whole country. Thirty-five years later, it’s at 0.6%. The problem is that many young minority faculty don’t make it. They do things that don’t get them promoted, while other faculty do only things that move them toward promotion. Yet, one thing you have to take into account in your “balance” is that you’re also becoming as an academic leader, not infrequently in an area of diversity. Even if you’re not a minority, academic leaders are looking to promote those people that are good scholars, but also have leadership. So, this is where assessing your balance between participation in activities outside of your research is important. And this is where you have to decide where your balance lies.

*M. DeBaun:* I want to make this concrete, because, sometimes, we give you these good ideas, but there not concrete. Christian interviewed at Vanderbilt. And he had a second-look day. I’ve never attended a second-look day at Vanderbilt for pediatrics. But every year for the last seven years, I’ve mentored a medical student at Meharry Medical College, a Historically Black Medical School, or medical students from Vanderbilt. And some of the medical students have gone on to get competitive funding for one-year pull-out competitive programs, such as the Doris Duke Charitable Foundation or the American Society for Hematology. So, you can see I’ve made a conscious decision. I have asked myself, how am I going to spend my effort to promote diversity and excellence? I’m not going to spend my effort on a feel-good moment. And I’ve rejected this approach for promotion for a long time [laughter]. But at the same time, I’ll move heaven and earth for the medical student who’s at a Meharry medical college across town to make sure that they get the same opportunity to pursue research that Vanderbilt students have at Vanderbilt have to work in my laboratory. You have to make a personal decision about what’s important to you, and then just go with it and don’t look over your shoulder.

*G. Flores:* **What is the single thing that you wish someone had told you when you were a beginning young investigator which would have substantially enhanced your success early on?**

*J. Raphael:* I would honestly ask for the resources that you need. Because once you take a position, it’s hard to suddenly negotiate and say, “I suddenly need a research assistant. Or I need a statistician. Or I need a research coordinator.” You have to become a good negotiator. And part of that is asking for the things that will allow you to be successful in your early life as a junior investigator. If you don’t, then that becomes very problematic. I wish someone had been more forceful in pointing that out to me in the beginning.

*J. Mendoza:* Finding out what your peers are getting in terms of competitive offers being made: what should you expect in a package, how much protected time, those kinds of things. That’s very important information. The institutions may not want you to have that because it will be to your advantage during negotiations. I wish there was a more systematic way of spreading that information around, but y’all are pretty smart, you might be able to figure out how to do that. Just having the information at hand is very important.

*J. Wang:* This is a very hard question. Because you can’t do a controlled experiment on life [laughter]. So it’s difficult to know if you’d do it differently. I wish somebody told me early on to focus—not to be involved in a million things, just to focus on a few things. Then, given limited time, limited resources, you’re probably going to be more productive. It depends on the personality of the person. If your talent is to go across, to make connections in different ways, then it’s probably the wrong advice. But in general, for academia, everyone tends to be very focused like the NIH institutes. All the reviewers are very focused, so to play the game, you have to be focused.

*E. Fuentes-Afflick:* It’s a really hard question. Personally, I don’t have many regrets. Starting off from a more negotiating perspective is good, but at that time, for me, it was clear that my department felt like they were taking a chance on me. And I didn’t have protected time. I didn’t have a lot of other resources. Try to be open and seek advice. We’re often reluctant to say, is this enough money? We’re often very private about that and for good reason. But to the extent that you can benchmark what you’re being offered will be reassuring and helpful. But you’re in a vulnerable negotiating position. Also, don’t go in thinking you’re an all-star and you’re going make an all-time record. You have to be just a little careful that you don’t overplay it.

*F. Jones:* For me, it’s getting outside of your comfort zone. When I first started, I was very focused, and stayed right within my lanes. But to really do something innovative and impactful, you have to get outside your comfort zone.

*M. DeBaun:* I was naively trusting of the system that all faculty recruits on the tenure track were given an equal shot for success and similar resources to begin their career. Not until I started to mentor other faculty did I realize tenure-track faculty were not all offered the same opportunities when they were recruited. In my first faculty position, I did not have a start-up package, even though my salary was fully funded, at greater than 90%, and I was on a tenure track. Perhaps the absence of a start-up funds was a good thing for my career. I had to focus my energy. I didn’t have the luxury of spending time on projects that were not going to lead to my next grant. But later in my career, I had a moment of clarity when I learned that other tenure-track junior faculty received start-up packages to improve the likelihood of success. The bottom line is, I would make sure that as a new faculty recruit, wherever you go, you receive more than a vote of confidence in your career, but also resources commensurate with your peers. You should also have access to close mentorship and resources on par with your peers to increase your likelihood of success.

*F. Mendoza:* I was the only minority faculty in 1981 in my whole medical school. And then I became a dean two years later. Looking back, what would have helped is to have been told, “learn how to write, and write well, and write often.” It took me a decade to do that. My first grant was in 1992. I’ve had 25 years of grant funding. I’m a better writer now. I don’t know that I’m a great writer, but I write better. I went into science because I didn’t like English [laughter]. But at the end of the day, I have learned that you are in academia what you write. And all these other things will happen if you learn how to write, write well, and write often. Moreover, today, there’s a need to speak for people that can’t speak, that is, for our communities that don’t get to be part of the public discourse. It’s through writing that we can help speak for them.

*G. Flores:* My advice would be, don’t let external milestones define you. By that, I mean, it’s easy to get caught up in, “if only I had had that award for career contributions, or if I only had that 200th publication, or if I only had that $5 million grant, then I would be successful, and I would be in heaven and achieved everything I needed.” It really should be about what John Wooden once said. John Wooden was probably the greatest college basketball coach ever. He broke all the records for consecutive NCAA championships, and someone asked him, “What drove you? Was it trying to break all the records?” He said, “No. Success is peace of mind, which is a direct result of self-satisfaction in knowing you did your best to become the best you are capable of becoming.” And I thought about that and I said, “Oh, come on, it’s got to be all those awards. That’s a great thing to have.” But it really is true. There’s no greater sense of satisfaction than fulfilling your own standards, saying, “I have a goal. I want to be an expert on unconscious bias, or disparities,” or whatever it is you’re working on, “so that I feel like I achieved the goals that I set for myself.”

Dr. DeBaun mentioned that a lot of people didn’t tell him what he should have known as a junior faculty coming in, so I want to ask the panel, **“How do you ask that question when you’re going to a new institution, especially if there are not a lot of people ahead of you? What should I be getting? How do you approach that?”**

*M. DeBaun:* There’s an inherent conflict, if the person you’re negotiating with is your mentor. You have to understand that you need an advocate outside of your mentor. And we’ve talked about these mentors: there’s a career mentor, there are peer mentors. And we haven’t said that enough. But you all will hopefully form peer mentorship, and you will be the best source of, “Well, what did you get? How did you negotiate this problem? How did you figure out, ‘hey, no, I don’t want more money for my start-up, I need a higher salary.'” Or you’ll find out that some people get their loans repaid. So you really do want to form these mentorship teams: career mentor in terms of your science, professional mentorship, someone in a different institution that you can have an offline discussion about your negotiation package, and then peer mentorship being people who are walking through the same steps you’re walking through so you can get their feedback.

*E. Fuentes-Afflick:* If you’re going to a new institution, try to use your network to say, “Do you know anyone at this place? Because I’m considering a job and I don’t have enough context to be able to interpret the offer that they’re making me.” Maybe that person can say, “My friend Jason is at Stanford. Let me contact Jason and see if he’d be willing to talk with you.” That’s also how you can leverage networks to help you. Not that Jason is going to advocate for you, but he might say, “Right now, everyone is getting 10% protected time*;* we’re just in a time of fiscal constraint and that’s really what we can offer.” So, it’s not different than what I’ve seen for other people, but it’s tough. With that information, you’ll feel, “Okay, my letter is consistent with what other people have been receiving.” So, use that network to try to plug into someone, but you also have to be comfortable. As Faye was saying, reaching out of your comfort zone, contacting someone who you don’t know, trusting that if someone connects you to someone, then you have to make the next step. People are not going to put it on a platter for you. Make sure that you feel comfortable. Also, reach out with a specific request. Again, not best friend forever and not going to bat for me, but just, I need a little more information so I can interpret this opportunity because I’m really excited about X, Y, and Z, but I have concerns about A, B, and C.

*F. Mendoza:* Know the rules of the academic game, and all your questions should focus on, how do I get promoted? You can get people to help you understand the process, but you have to know clearly what it takes to go from assistant to associate professor. You should ask your division chief, the person that’s going to be hiring you, or the person that you’re working for in that institution. You should talk to the department chair and sometimes also the dean of academic affairs. If all three points of view on promotion line up, then you will know what exactly you need to get promoted to associate professor. I’ve seen situations, however, where each of those people relate different things. In that scenario, you’re stuck. A few years ago, a faculty member who was a neurosurgeon said to me, “My department chair said that I should do a hundred cases a year and teach, and now I’m going to be up for tenure, because he thinks I’ll get promoted.” I told him that I was concerned because he had not published. This a case where someone did what his chairman said, but the information he received about promotion requirements was not totally accurate, and consequently, he was left in the difficult position of possibly not being promoted. So, just make sure you know what you need to do to be promoted, because then, things are more likely to fall into place for you and your career.

*F. Jones:* The other important part that you need to look at for any institution that you’re going to is what’s the climate’s like, because it may all look really nice on the surface with people getting promoted, but when you look at the climate hard, are the underrepresented faculty getting promoted at the same rate as everybody else. All of those things are important, so dig that information out. One simple thing I did several years ago is to actually talk to people who left the institution. That can be very informative, because sometimes those individuals will say, “the reason I left was to be closer to family,” or something else. Or, they may say, “this is something very specific to this institution that I did not like.” Sometimes, you have to take those things with a grain of salt. But this is a business decision, so if I’m going to invest here, what would I do? Talk to the people at the company. I’d talk to people who’ve left the company, so that you cover all the bases and get the information you need. The other thing is, you get in front of that person that’s your mentor, they’re not looking at you as their mentee right now. They’re looking at you as an item on a spreadsheet that they want for the cheapest price possible, and you’re like, “but you’re my mentor,” or, “you’ve been this amazing person.” That’s gone in their mind, and your feelings will get hurt. You will compromise your future stability if you look at them as your mentor in that moment. So, if you’re going to negotiate with them, it’s got to be all business.

*J. Wang:* Negotiating with any institution, it’s like buying a house. If you’re going to move to an area and buy a house, you want to know what the average housing price is. Those data are published for every single specialty by region: average salaries for assistant and associate professor. So, you should get hold of that, or the department chairs have them. The other thing is that you should know what you have. If you go to go buy a house, you should know how much cash you have, how many loans you have, and what your credit score is. It’s the same thing with a job: you should know if you’re bringing in funding, if you’re going to need support, and if you have a good track record that’s almost like your credit score. You don’t walk into a negotiation without knowing what the average market conditions are and what you have to bring to the table. And after you do that, it’s always good to have an advocate, so you don’t directly go and ask. Maybe you asked for an advocate who could answer on your behalf. So, it’s not a yes or no question, but, “how can I do this?” There’s a book on getting to yes, and one on getting past no. So, just read those two books. [laughter].

## Conclusions

This is the first published practical guide (to our knowledge) on keys to academic success for URM young investigators. The NAC panel of leading senior pediatric researchers identified the following keys to success for URM investigators: having multiple mentors, writing prolifically, being strategic about your financial situation, having a passion for you research topic, confidence about your capacity for success, and being tenaciously persistent. Advice on ensuring the best possible mentorship included having different mentors for different purposes, taking an active role in getting what you need from mentors, choosing mentors who have track records of success, having mentors who are invested in you, identifying mentors who are a good fit, having a mentor who also is a sponsor (able to connect you to other key leaders in the field), and having a member of the mentorship team who is a coach.

NAC panelists’ advice on dealing with families who do not want you to care for their child because of your race/ethnicity included seeking to understand the reasons, transferring the patient to another institution, having a faculty forum about the issue, making sure the patient gets needed care, taking care of yourself emotionally, debriefing with colleagues, and turning it into a positive by writing about it, teaching others, and developing a curriculum. Panelists’ guidance on maximizing funding success included seeking non-traditional funding streams, building a portfolio of small grants, leveraging funding from mentors, writing many proposals, working with partners, networking, strategically analyzing the funder pay lines, establishing a publication track record, negotiating protected time and resources in new jobs, and participating in study sections.

Regarding balancing committee work with having enough time to advance one’s research and career, the panel advised using these opportunities to generate scholarly products, adding such committee work to your CV, choosing committees strategically with the help of a mentor, joining committees in the context of being an expert, developing a triage process based on interests and being politically savvy, balancing commitments with an eye towards promotion, and prioritizing activities that promote diversity and excellence. The most important things that panel members wished someone had told them when they started out as a young investigator included asking for all of the needed resources, identifying what others negotiated for to determine the components of competitive job offers, having focus, being open and seeking advice, getting outside your comfort zone, learning how to write well and often, and focusing on fulfilling your own standards of excellence rather than achieving external milestones and validation.

## Data Availability

All data generated are included in this published article.
